# Stereotactic Body Radiation Therapy for Prostate Cancer: An Institutional Experience Using MRI-guided Treatment Planning

**DOI:** 10.7759/cureus.2590

**Published:** 2018-05-08

**Authors:** Nabila L Waheed, Alison K Yoder, Renae D Van Wyhe, Steven L Carpenter

**Affiliations:** 1 Radiation Oncology, Baylor College of Medicine; 2 Medical Student, Baylor College of Medicine

**Keywords:** prostate cancer, sbrt, mri treatment planning

## Abstract

With 222,500 new cases estimated for 2017, prostate cancer makes up approximately 10% of all new cancer diagnoses in the United States and is the third most common cancer after breast and lung cancer. In 2013, the American Society of Radiation Oncology (ASTRO) policy model recognized that stereotactic body radiation therapy (SBRT) may be used as an alternative to standard treatment modalities, i.e. intensity modulated radiation therapy (IMRT), to treat prostate cancer. In this study, we report outcomes for a cohort of 30 patients with prostate cancer treated with SBRT at our institution. We also describe, in detail, the technical aspects of SBRT planning and delivery for these patients, specifically the use of MRI in determining treatment volumes and detecting gross lesions.

After institutional review board (IRB) approval, a retrospective analysis was done of 30 males with the diagnosis of prostate cancer treated in the Department of Radiation Oncology at the Baylor College of Medicine between January 2011 and June 2016. All patients received image-guided SBRT. Treatment planning was performed using a non-contrast computed tomography (CT) scan as well as a contrast thin-slice open MRI with the patient in the treatment position. Patient comparisons were done using the Mann-Whitney U, Fishers Exact, and Kaplan-Meier tests.

Thirty patients were treated between January 2011 and June 2016. Twenty-six had follow-up data available and were included in the analysis. Median follow-up was 32 months (range 2-72 months). Mean and median ages at diagnosis were both 68.5 years. A total of 64% of the patients had foci on magnetic resonance imaging (MRI) or a palpable nodule on an exam. The median prostate-specific antigen (PSA) at diagnosis was 7.35 ng/mL (range 2.8-13), and the median PSA nadir after treatment was 0.4 ng/mL (range 0.01-4.5). The biochemical disease-free recurrence rate per Phoenix definition was 96%, with only one patient experiencing a biochemical recurrence four years after treatment. The patient with a recurrence was T2c, high-intermediate risk with a Gleason score of 7(3+4). He had a focus visible on MRI. Overall survival was 96%, with the only patient death unrelated to his prostate cancer. There was no statistical significance associated with recurrence and nodule on MRI (p=0.318), T-stage (p=0.222), Gleason score (p=0.890), risk group (p=0.654), age (p=0.692), or race (p=0.509). There were no grade three or four acute or long-term toxicities.

SBRT of the prostate is an effective method for treating prostate cancer. We saw excellent PSA control and minimal acute or long-term toxicities after a median of three years of follow-up.

## Introduction

With 222,500 new cases estimated for 2017, prostate cancer makes up approximately 10% of all new cancer diagnoses in the United States and is the third most common cancer after breast and lung cancer [1]. The standard of care for prostate cancer depends largely on the risk group classifying the aggressiveness and extent of disease: low, favorable intermediate, unfavorable intermediate, or high risk [2]. According to the National Comprehensive Cancer Network (NCCN) Guidelines Version 2.2017, recommendations for low-risk and intermediate-risk prostate cancers include external beam radiation therapy (EBRT) with or without androgen deprivation therapy and/or brachytherapy adjuvant to these modalities, surgery, or observation depending on a 10-year life expectancy (PROS-4). Recently, however, the treatment of low- and intermediate-risk prostate cancer with a highly hypofractionated five-fraction regimen has been described [3-5]. In 2013, the American Society of Radiation Oncology (ASTRO) policy model recognized that stereotactic body radiation therapy (SBRT) may be used as an alternative to standard treatment modalities, i.e. intensity modulated radiation therapy (IMRT), to treat prostate cancer. NCCN Guidelines Version 2.2014 reiterated this suggestion.

The rationale for SBRT can be explained by tumor biology. Namely, prostate cancer grows slowly, evidenced by its low α/β ratio. As such, it is highly sensitive to large fraction size. Thus, a large number of small fractions, for example, 2 Gy given in 38-39 fractions or 1.8 Gy given in 45-48 fractions, may not be the ideal approach [6]. In several reports, SBRT to 35 or 36.25 Gy in five fractions has been reported to be an acceptable substitution as a treatment modality for prostate cancer over IMRT or high dose rate (HDR) brachytherapy for low- and intermediate-risk cases, especially given its low cost, shortened treatment regimen, noninvasiveness, geometric accuracy, and potential for reduced toxicities [3-5,7-8]. As such, it is of paramount importance to describe the technical aspects of SBRT with the corresponding clinical outcomes as they become available.

In this study, we report outcomes for a cohort of 30 patients with prostate cancer treated with SBRT at our institution. We also describe, in detail, the technical aspects of SBRT planning and delivery for these patients, specifically the use of magnetic resonance imaging (MRI) in determining treatment volumes and detecting gross lesions. After a median of three years of follow-up, we report minimal acute or late toxicity and excellent PSA control in patients who underwent SBRT to the prostate.

## Case presentation

Materials and methods

Patient Population

Institutional review board (IRB) approval was obtained for this single-institution retrospective analysis of patient charts and radiation treatment records. A retrospective analysis was done of 30 males with a diagnosis of prostate cancer treated at the department of radiation oncology at the Baylor College of Medicine between January 2011 and June 2016. Twenty-six of these patients have follow-up data available and were included in the analysis.

All patients were diagnosed with biopsy-proven non-metastatic prostate cancer. Patients were categorized by the risk group of prostate cancer, following the standard four risk groups as per NCCN 2.2014. All patients were treated definitively with SBRT using the CyberKnife (Accuray, California, US) platform. Among our 26 patients, seven were low-risk, 17 intermediate risk, one high risk, and one had a recurrence after undergoing cryotherapy.

Treatment Details: Fiducial Placement

Approximately one week prior to treatment planning, four gold fiducial seeds were placed in most patients to allow for motion tracking during treatment. This was done with the transgluteal approach using computed tomography (CT) guidance by an interventional radiologist. The transgluteal approach was used in order to avoid the penetration of the rectum by the implantation needle. A few patients had fiducials placed by the treating radiation oncologist using a transperineal approach. The seeds were implanted in the prostate, with a minimum of 20-mm spacing from all other fiducials.

Treatment Details: Localization Imaging with MRI and CT Simulation

The patients were asked to complete a bowel prep that included Dulcolax (Boehringer Ingelheim, Ingelheim, Germany) the day prior to simulation. In addition, a Fleets enema was used at home prior to simulation or treatment (C.B. Fleet Company, Inc., Lynchburg, Virginia).

After allowing approximately a week for possible seed migration, treatment planning was performed using a CT scan (15-mm cuts) as well as a thin-slice MRI on a 1.0 Tesla open MRI (with imaging characteristics of 1.5 Tesla). The MRI was done with intravenous (IV) contrast in the treatment position via a vac-lock bag. Most patients were treated with an empty bladder. Patients were placed in the supine position in the immobilization device. Contrast MRI and a non-contrast CT scan of the pelvis from above the iliac crest to below the ischium were performed.

Treatment Details: Target Volume and Organ at Risk (OAR) Contouring

All pretreatment imaging was performed with the patient in the same position as was used for his treatment delivery. For low-risk patients, just the prostate made up the gross target volume (GTV). For intermediate- to high-risk patients who had a Gleason Score of greater than 6 or a prostate-specific antigen (PSA) of greater than 10 ng/ml, the proximal half of the seminal vesicles was added to the GTV. GTV was identified in the transverse, sagittal, and coronal orientation. After the GTV was delineated, a margin was added to create the clinical target volume (CTV). For low- and intermediate-risk patients, the margin was extended 5 mm on all sides except for posteriorly (by the rectum) where a 3-mm margin was used. All patients had the bladder, prostate, rectum, seminal vesicles, urethra, testicles, sigmoid colon, and penile bulb contoured.

Treatment Details: Dose Prescription

All patients received image-guided SBRT using the CyberKnife with MultiPlan (Accuray) inverse treatment planning and motion tracking throughout treatment based on internal fiducials.

The patients received a total dose of 36.25 Gy in five fractions of 7.25 Gy to cover at least 96% of the CTV. The number of beams ranged from 140 to 170. Areas of T2 hypodensities were contoured as gross disease. These areas received dose painted simultaneous integrated boost to approximately 40 Gy in five fractions.

Treatments were performed on five non-consecutive days. In the morning before each treatment, patients completed a bowel prep that included Dulcolax (Boehringer Ingelheim, Ingelheim, Germany) and a Fleet enema (C.B. Fleet Company, Inc., Lynchburg, Virginia).

Treatment Details: Dose Constraints for OARs 

The mean D50 to the bladder and rectum was 43% and 41% of the prescribed dose, respectively. The Volume of the rectum receiving 36 Gy was limited to 1 cc, and the volume of the bladder receiving a maximum of 37 Gy was limited to 10 cc. Dose constraints for other organs at risk are as follows: V47 Gy for the prostatic urethra was limited to 20%, V37 Gy of the membranous urethra was limited to 50%, the volume of the sigmoid receiving a dose of 30 Gy was limited to 1 cc, and the penile bulb volume receiving 29.5Gy was limited to 50% of the overall volume.

Treatment planning and execution

Orthogonal 120 kV X-ray image pairs were obtained throughout treatment for use in motion tracking. The real-time prostate position was locked on by the relative fiducial position on the X-rays. Prostate movements in all directions, yaw, pitch roll, X, Y, and Z, were tracked and corrected for all patients in real time.

Typical treatments consist of about 100-120 non-coplanar beams with total treatment averaging 40-60 minutes. Imaging correction was done every five to seven beams (about every 30–90s).

Follow-up Evaluation

Follow-up goals included a complete physical examination at the completion of external beam radiation for all patients. Patients were also examined at the time of follow-up six to 10 weeks after completion of their radiation treatment, every three months thereafter for the following year, and every six months after that for two years. Treatment response was assessed for all patients by performing a clinical exam and PSA at the time of follow-up visits.

Analysis

The PSA nadir was defined as the lowest PSA value following SBRT. Biochemical failure (BF) was assessed using the nadir + 2 (Phoenix) definition. Toxicity was assessed using Common Terminology Criteria for Adverse Events (CTCAE) 4.03; acute toxicity occurred within three months and late toxicity greater than three months following treatment.

Pretreatment factors were assessed for each patient, which included the standard risk factors of initial PSA, Gleason score, and clinical stage. Patients were stratified into four risk groups as per NCCN 2.2014. To explore group heterogeneity, we separated our previous intermediate risk group into favorable intermediate if there was one intermediate risk factor (Gleason 7, clinical stage T2b/c, or PSA > 10 ng/dL) or unfavorable intermediate if there was more than one intermediate risk factor. For the purpose of further analysis, the unfavorable intermediate group was then combined with the high-risk group as per NCCN version 2.2014.

Kaplan–Meier estimates of freedom from biochemical failure (FFBF) were used to describe the patients overall, and comparisons were accomplished using Mann-Whitney U or Fishers Exact tests when appropriate.

Results

Thirty patients were treated between January 2011 and June 2016. Twenty-six of these patients had follow-up data available and were included in the analysis. Median follow -up was 32 months (range 2-72 months). Mean and median ages at diagnosis were both 68.5 years. A total of 62% (n=16) of patients had foci on MRI or a palpable nodule on an exam. Median PSA at diagnosis was 7.35 ng/mL (range 2.8-13) and median PSA nadir after treatment was 0.4 ng/mL (range 0.01-4.5). Two patients experienced a PSA bounce after treatment, both with a return to pre-bounce levels in less than 12 months (Table 1).

**Table 1:** Demographics of patients receiving SBRT to the prostate * Mann-Whitney Test ** Fisher's Exact Test ^Ɨ^ Combined with high-intermediate group for analysis PTV: planning target volume

	Number of Patients	Biochemical Recurrence	p-value
	n (%) median (range) mean (sd)	n	
Age at Diagnosis			
Median (range)	68.5 (55-84)		p=0.692 *
50-59	3 (11.5%)	0	
60-69	12 (26.2%)	0	
70-79	9 (34.6%)	1	
80-89	2 (7.8%)	0	
Race			p=0.546 **
Black	5 (19.2%)	0	
White	21 (80.8%)	1	
PSA Level at Treatment	(ng/mL)		p=0.308 *
Mean (sd)	7.63 (2.97)		
Median (range)	7.35 (2.8-13)		
PSA Level at Diagnosis			p >0.99 **
<4 ng/mL	2 (7.8%)	0	
4-10 ng/mL	20 (76.9%)	1	
>10-20 ng/mL	4 (15.4%)	0	
Risk Category			p >0.99 **
Low	7 (26.9%)	0	
Low Intermediate	9 (34.6%)	0	
High Intermediate	8 (30.8%)	1	
High^Ɨ^	1 (3.8%)	0	
Recurrence after cryo	1 (3.8%)	0	
Clinical Stage			
T1a	1 (3.8%)	0	p=0.154 **
T1c	18 (69.2%)	0	
T2a	4 (15.4%)	0	
T2c	2 (7.8%)	1	
Recurrence after cryo	1 (3.8%)	0	
Gleason Score			p >0.99 **
6	7 (26.9%)	0	
7 (3+4)	15 (57.7%)	1	
7 (4+3)	2 (7.8%)	0	
8	1 (3.8%)	0	
Recurrence	1 (3.8%)	0	
Radiation Treatment			
Mean minimum PTV dose (sd)	3144 (+/- 238)		p=0.846 *
Mean minimum prostate dose (sd)	3578 (+/- 204)		p=0.692 *
Foci on MRI			p=0.615 **
Yes	16 (61.5%)	1	
No	10 (38.5%)	0	

The biochemical disease-free recurrence rate per Phoenix definition was 96%, with only one patient experiencing a biochemical recurrence four years after treatment. The patient with a recurrence was T2c, high-intermediate risk with a Gleason score of 7(3+4). He had a focus visible on MRI. Overall survival was 96%, with one patient death that was unrelated to his prostate cancer.

**Figure 1:**
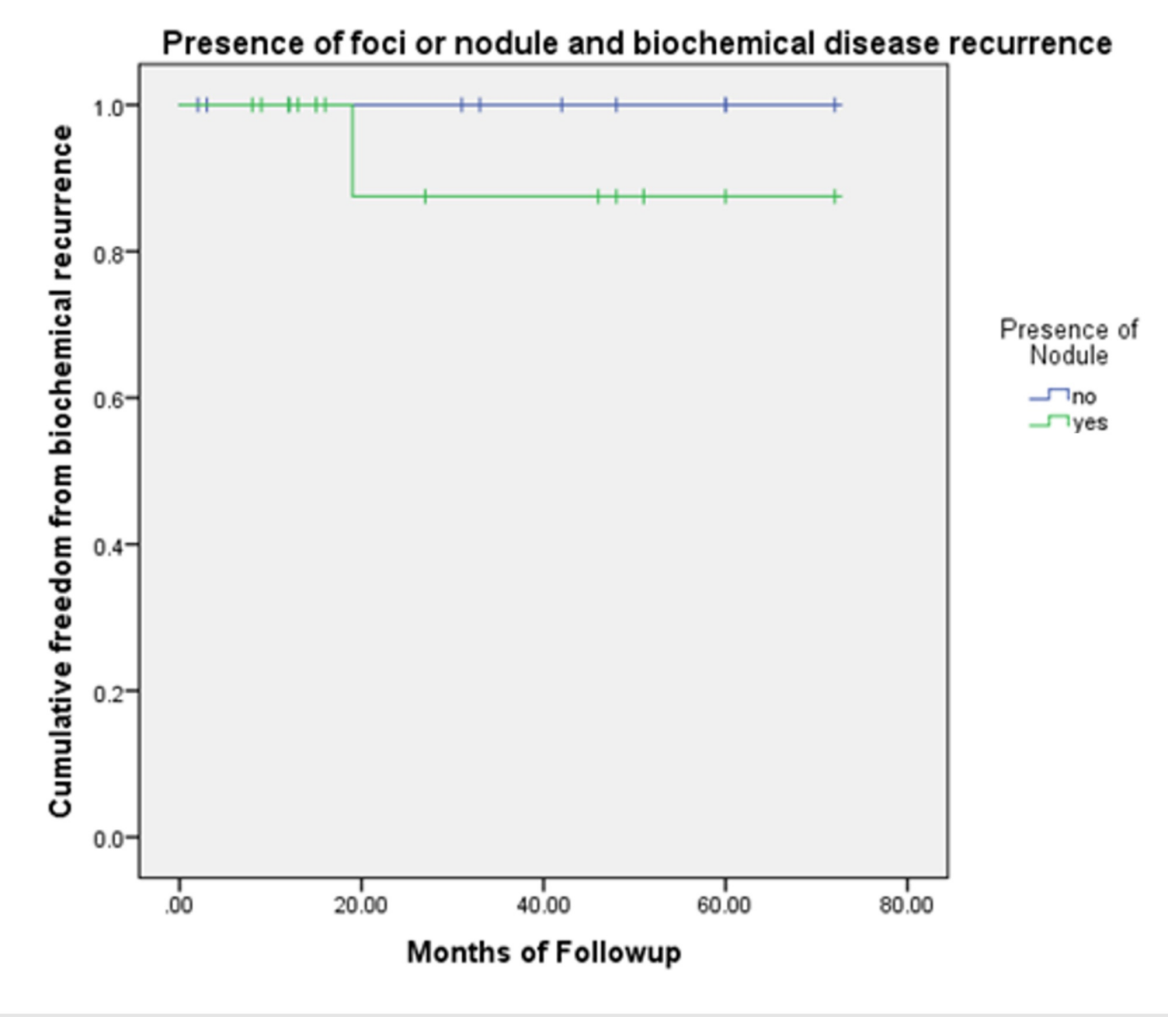
Kaplan-Meier curve comparing the presence of a nodule on MRI and biochemical disease recurrence

**Figure 2:**
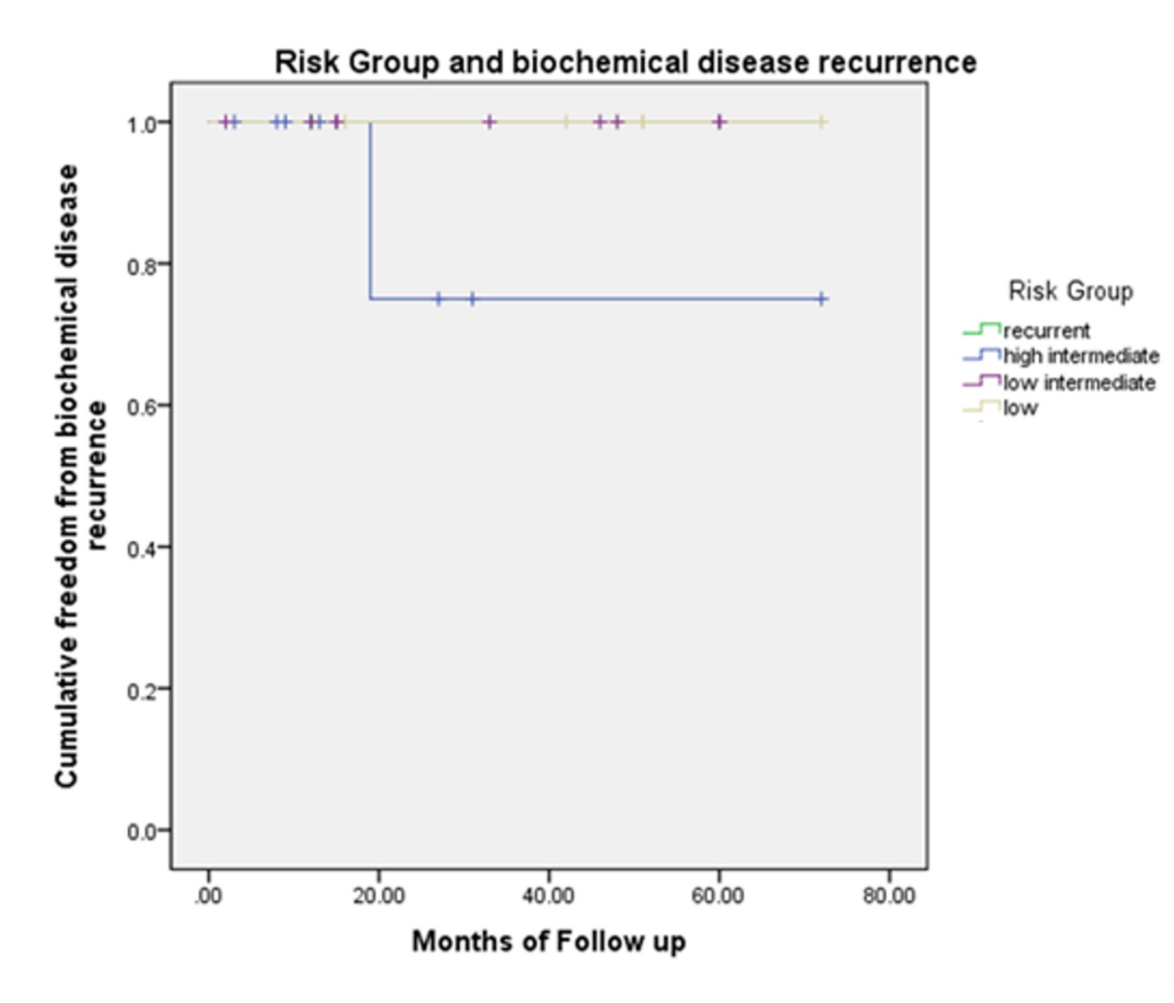
Kaplan-Meier curve comparing risk group and biochemical disease recurrence

**Figure 3:**
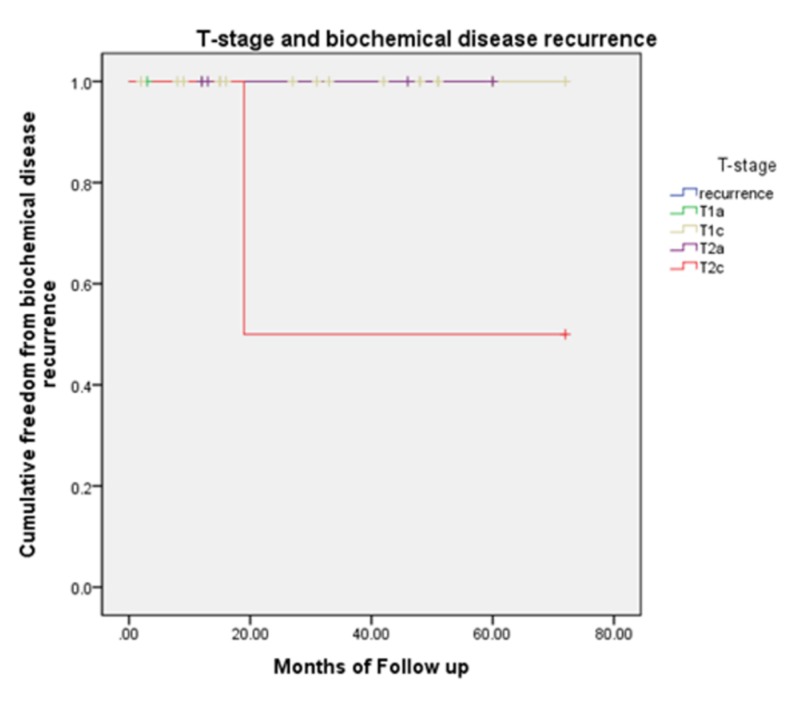
Kaplan-Meier curve comparing T-stage and biochemical disease recurrence

There was no statistical significance associated with recurrence and nodule on MRI (p=0.318), T-stage (p=0.222), Gleason score (p=0.890), risk group (p=0.654), age (p=0.692), or race (p=0.509) using the Mann Whitney U and Fishers Exact tests. Kaplan-Meier curves for the presence of foci/nodule, risk group, and T-stage are included (Figures 1-3).

**Figure 4:**
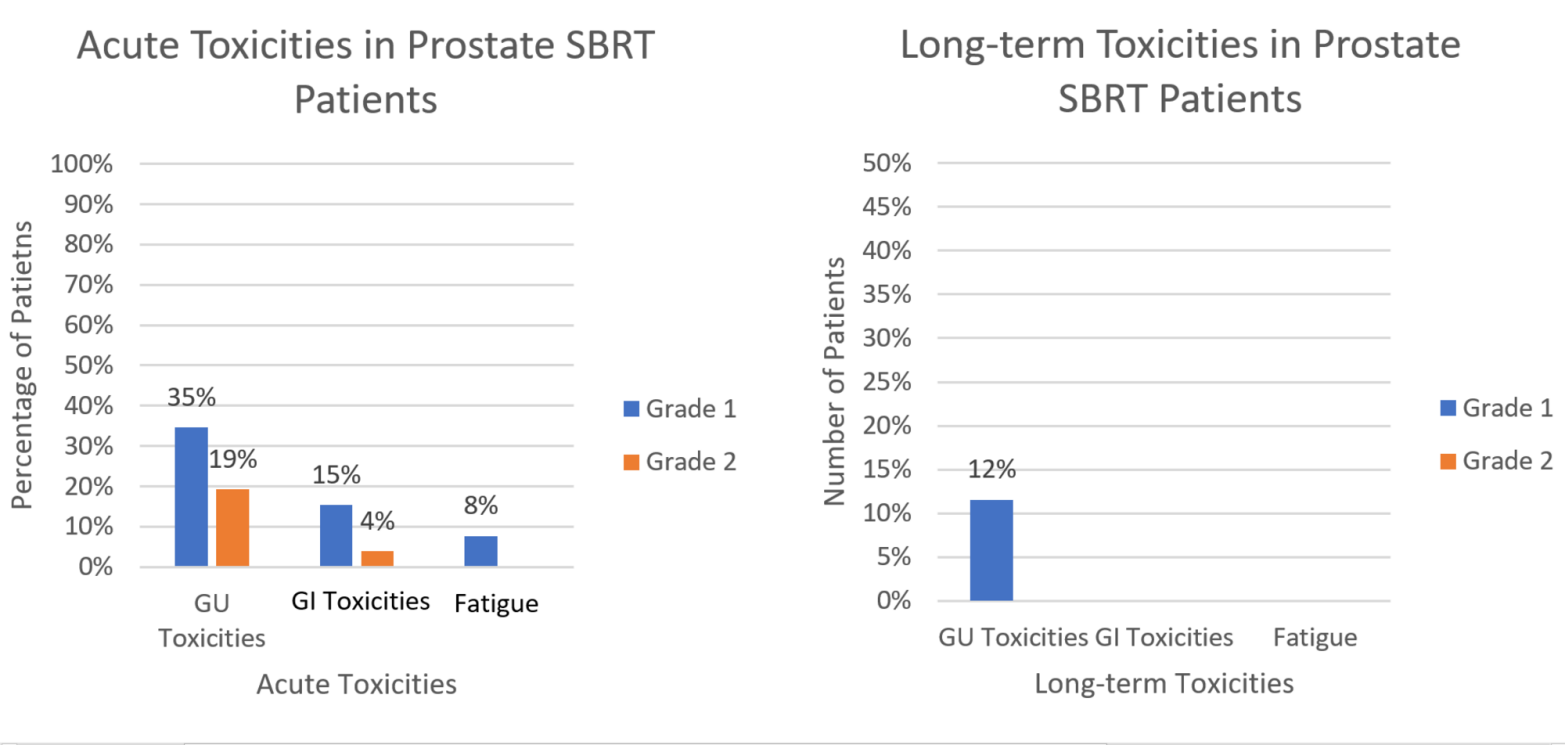
Acute and long-term toxicities in patients treated with SBRT to the prostate SBRT: stereotactic body radiotherapy; GU: genitourinary; GI: gastrointestinal

The most common complaint was mild dysuria or frequency not requiring medication. Five patients did require medication during treatment, two for urinary frequency and three for urinary retention. One patient with urinary retention required a break-in treatment, but the retention resolved with steroids and Flomax without the requirement of catheter placement. One patient with urinary frequency also had proctitis that resolved with steroids. There were no grade three or four acute toxicities from treatment. The most common complaint after treatment ended was mild dysuria and frequency, none requiring medication (Figure 4).

**Figure 5:**
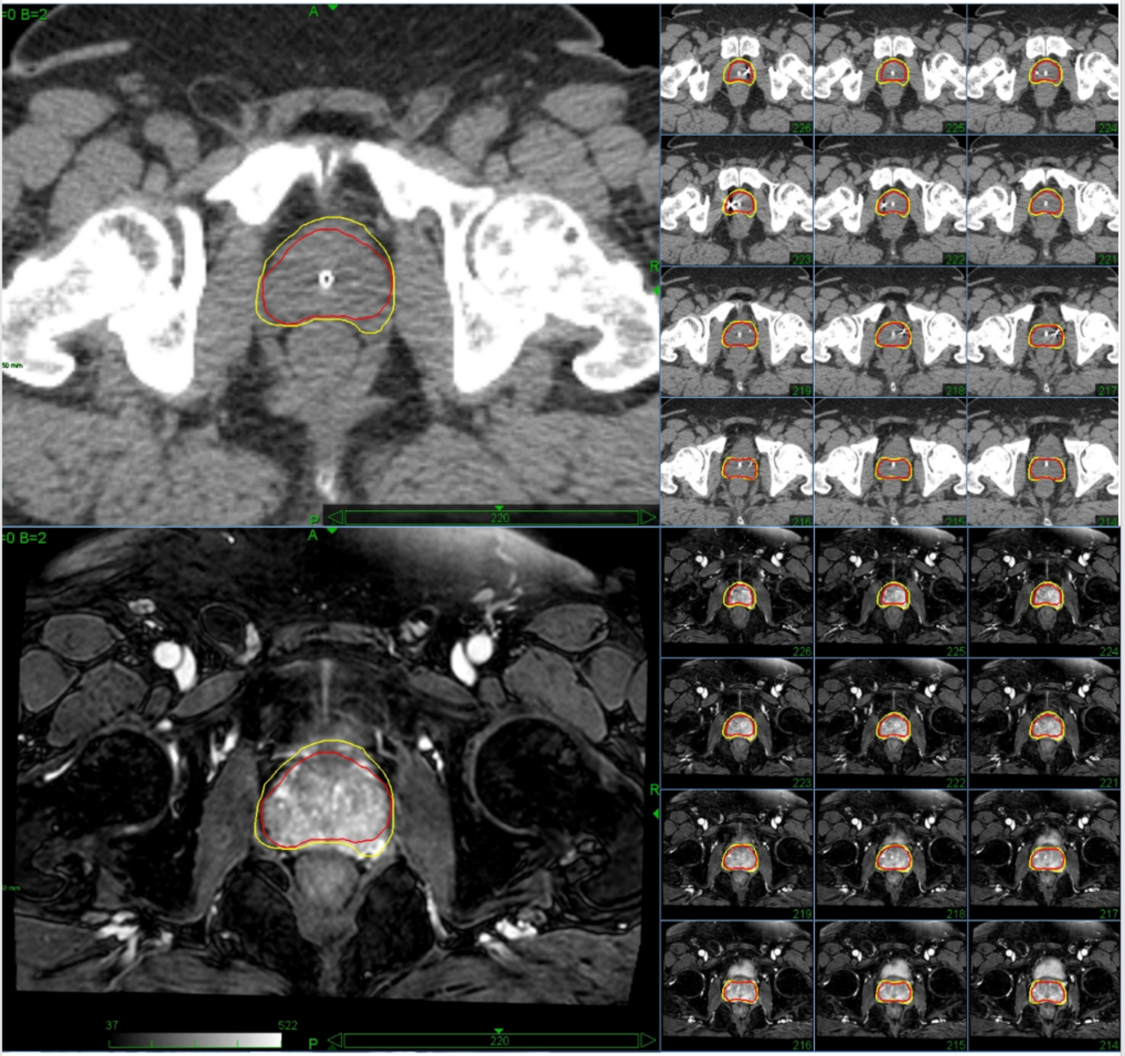
Comparing the contours of the prostate using CT (top) vs. MRI (bottom) Yellow = Contour of the prostate using computed tomography (CT) Red = Contour of the prostate using open magnetic resonance imaging (MRI)

As seen in Figure 5, the use of MRI allowed for improved precision of contouring, as we were able to identify the prostate apex, penile bulb, and neurovascular bundle on MRI that are not visualizable on CT. This resulted in a reduced volume of the clinical target volume (CTV). In the example shown, the CTV volume contoured was reduced from 74.6 cc on CT to 49.5 cc on MRI.

## Discussion

There was a high rate of disease control for patients treated using SBRT, with 96% remaining biochemically free of disease. Patients tolerated the treatment well, with only one break in treatment required for urinary retention. All patients have continued to report minimal side effects, with no long-term grade three or four genitourinary (GU) or gastrointestinal (GI) toxicities.

These results are comparable to previous studies and suggest that SBRT is comparable to HDR brachytherapy and possibly superior to traditional IMRT for prostate cancer disease control [8]. Cyberknife SBRT is non-invasive and does not require sedation, which renders it favorable to HDR for many patients. In addition, most studies, including ours, have not demonstrated toxicity profiles to be significantly worse than traditional therapy at the standard dose of 36.25 Gy [9]. This study does not indicate a need for dose escalation, which has shown increased toxicities in the past.

Our procedure differed from most institutions in that we used an open-MRI for simulation as opposed to CT alone. This allowed us to reduce the total volume being targeted, as well as to identify and avoid nearby structures, including the penile bulb, neurovascular bundle, and prostate apex. MRI has been used previously in treatment planning for prostate cancer, with positive results. The use of MRI for treatment planning allows for a better visualization of the prostate as well as surrounding structures due to enhanced soft-tissue contrast. This method can increase the precision of contouring, which can reduce the volume contoured and the side effects seen from treatment [10]. For the patient shown, the CTV volume was decreased by 34.6% using MRI as opposed to traditional CT for contouring. The use of MRI also gives the ability to see the tumor nodule, allowing for the capability to dose-paint the neoplasm itself.

In addition, this procedure differed from previous reports in the use of open-MRI as opposed to traditional closed MRI. This allowed us to put patients in the treatment position in vac-lock, with the bladder and bowel in the same fill state and without an endorectal coil during their planning MRI. This improves reproducibility and registration during contouring. There have been concerns over uncertainties in organ position when using MRI to plan prostate therapy [10], but the use of open MRI reduces these uncertainties, as the patient is already in treatment position during simulation.

Overall, these findings build on the literature that SBRT is an effective alternative for prostate cancer treatment. Further randomized studies are needed to determine the most-effective method for treating prostate cancer. In addition, the utility of open MRI for radiation planning has significant promise and should be further explored as a method for improving organ delineation and reducing treatment toxicities.

## Conclusions

SBRT of the prostate is an effective method for treating prostate cancer. We saw excellent PSA control and minimal acute or long-term toxicities after a median of three years of follow-up. The use of open MRI with the patient in the treatment position during planning allowed for an improved visualization of the prostate and an increased precision of contouring.
